# A data set from flash X-ray imaging of carboxysomes

**DOI:** 10.1038/sdata.2016.61

**Published:** 2016-08-01

**Authors:** Max F. Hantke, Dirk Hasse, Tomas Ekeberg, Katja John, Martin Svenda, Duane Loh, Andrew V. Martin, Nicusor Timneanu, Daniel S.D. Larsson, Gijs van der Schot, Gunilla H. Carlsson, Margareta Ingelman, Jakob Andreasson, Daniel Westphal, Bianca Iwan, Charlotte Uetrecht, Johan Bielecki, Mengning Liang, Francesco Stellato, Daniel P. DePonte, Sadia Bari, Robert Hartmann, Nils Kimmel, Richard A. Kirian, M. Marvin Seibert, Kerstin Mühlig, Sebastian Schorb, Ken Ferguson, Christoph Bostedt, Sebastian Carron, John D. Bozek, Daniel Rolles, Artem Rudenko, Lutz Foucar, Sascha W. Epp, Henry N. Chapman, Anton Barty, Inger Andersson, Janos Hajdu, Filipe R.N.C. Maia

**Affiliations:** 1 Department of Cell and Molecular Biology, Laboratory of Molecular Biophysics, Uppsala University, Husargatan 3 (Box 596), Uppsala SE-751 24, Sweden; 2 Centre for BioImaging Sciences, National University of Singapore, 14 Science Drive 4, Singapore 117543, Singapore; 3 ARC Centre of Excellence for Advanced Molecular Imaging, School of Physics, The University of Melbourne, Victoria 3010, Australia; 4 Department of Physics and Astronomy, Uppsala University, Lägerhyddsvägen 1, Box 516, Uppsala SE- 751 20, Sweden; 5 ELI beamlines, Institute of Physics, Academy of Sciences of the Czech Republic, Na Slovance 2, Prague 18221, Czech Republic; 6 Center for Free-Electron Laser Science, DESY, Notkestrasse 85, Hamburg 22607, Germany; 7 I.N.F.N. and Physics Department, University of Rome ‘Tor Vergata’, Via della Ricerca Scientifica 1, Rome 00133, Italy; 8 LCLS, SLAC National Accelerator Laboratory, 2575 Sand Hill Road, Menlo Park, California 94025, USA; 9 European XFEL GmbH, Albert-Einstein-Ring 19, Hamburg 22761, Germany; 10 Deutsches Elektronen-Synchrotron DESY, Notkestr. 85, Hamburg 22607, Germany; 11 PNSensor GmbH, Römerstrasse 28, München 80803, Germany; 12 Max Planck Institute for Extraterrestrial Physics, Giessenbachstrasse, Garching 85741, Germany; 13 Department of Physics, J.R. Macdonald Laboratory, Kansas State University, Cardwell Hall, Manhattan, Kansas 66506, USA; 14 Max Planck Institute for Medical Research, Jahnstraße 29, Heidelberg 69120, Germany; 15 Max Planck Advanced Study Group at the Center for Free-Electron Laser Science (CFEL), Notkestraße 85, Hamburg 22607, Germany; 16 Max Planck Institute for Nuclear Physics, Saupfercheckweg 1, Heidelberg 69117, Germany; 17 Max Planck Institute for the Structure and Dynamics of Matter, Luruper Chaussee 149, Hamburg 22761, Germany; 18 NERSC, Lawrence Berkeley National Laboratory, Berkeley, California 94720, USA

**Keywords:** Optics and photonics, Biophysics

## Abstract

Ultra-intense femtosecond X-ray pulses from X-ray lasers permit structural studies on single particles and biomolecules without crystals. We present a large data set on inherently heterogeneous, polyhedral carboxysome particles. Carboxysomes are cell organelles that vary in size and facilitate up to 40% of Earth’s carbon fixation by cyanobacteria and certain proteobacteria. Variation in size hinders crystallization. Carboxysomes appear icosahedral in the electron microscope. A protein shell encapsulates a large number of Rubisco molecules in paracrystalline arrays inside the organelle. We used carboxysomes with a mean diameter of 115±26 nm from *Halothiobacillus neapolitanus*. A new aerosol sample-injector allowed us to record 70,000 low-noise diffraction patterns in 12 min. Every diffraction pattern is a unique structure measurement and high-throughput imaging allows sampling the space of structural variability. The different structures can be separated and phased directly from the diffraction data and open a way for accurate, high-throughput studies on structures and structural heterogeneity in biology and elsewhere.

## Background & Summary

X-ray free-electron lasers generate ultra-short and extremely bright coherent X-ray pulses. Such pulses can be used to produce diffraction patterns from a single particle before it is destroyed due to radiation-induced processes^1–3^, as demonstrated in recent experiments^2,4–6^. Sample heterogeneity, inherent in biology, interferes with crystallisation leading to systematic gaps of knowledge in structural biology. This prevents most large biological entities (e.g., cell organelles, large virus particles, flexible protein complexes) from being crystallized, and even when crystallization is successful only the reproducible regions can be resolved. Flash X-ray imaging (FXI) does not rely on crystallisation and thus has the potential to study structures of single particles and biomolecules that exhibit heterogeneity. High-throughput FXI is needed to measure three-dimensional structures, to achieve high-resolution and may even allow solving flexible structures. It has been shown that the concept of ‘diffraction before destruction’^1^ gives a significant advantage for resolving radiation sensitive material^7,8^. Although simple in concept, collecting sufficient high-quality diffraction patterns and separating the different structures present in the data is challenging.

**Figure 1:**
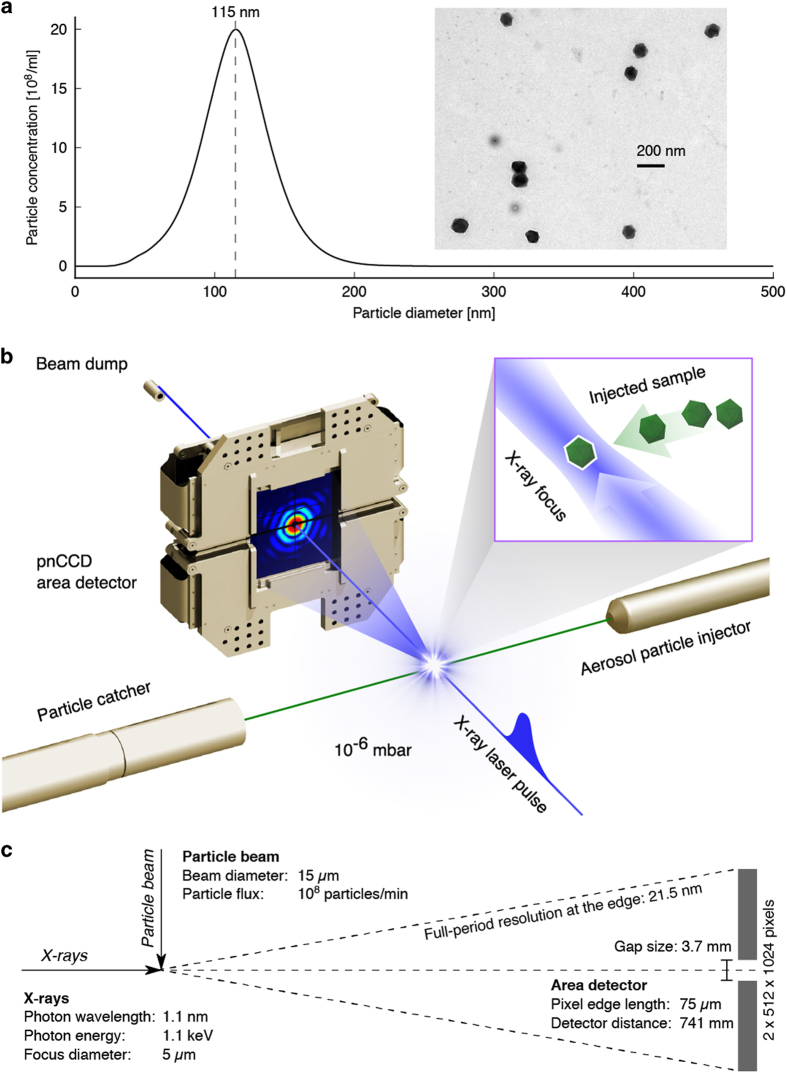
The experimental set up to image aerosolised carboxysomes. Figure adapted from ref. 5. (**a**) Size distribution of purified carboxysome particles in solution prior to aerosolisation. The particle size was measured by nanoparticle tracking analysis (NTA). The single peak with 115±26 nm diameter, showing a broad Gaussian size distribution. The insert shows an electron micrograph of isolated carboxysomes. (**b**) An aerosol particle injector delivers a focused stream of carboxysomes into the beam of the X-ray laser. Particles are hit in unknown orientations by the X-ray pulses. Diffraction patterns are recorded with a pnCCD X-ray area detector downstream of the interaction region. The intense primary beam passes through a gap between the two detector halves and is absorbed in a beam dump at a distance behind the detectors. (**c**) Experimental parameters and geometry.

We publish here an FXI dataset on aerosolised particles from a suspension of carboxysomes that were purified from the proteobacterium Halothiobacilus neapolitanus. This is the same data set used in the publication of Hantke *et al.* ^5^. Carboxysomes are polyhedral cell organelles that vary in size and in shape (Fig. 1a). The purified carboxysomes were complementary studied prior to injection by single-particle tracking analysis and electron microscopy (Fig. 1a). The size range is wide and peaks at 115 nm and the carboxysomes share an icosahedral appearance.

The experimental design is depicted in Fig. 1b,c. Aerosolised carboxysomes are injected into the X-ray pulse train of the Linac Coherent Light Source (LCLS) and diffraction patterns of isolated particles are recorded with an area detector. The direct beam passes through a narrow gap between the two detector halves and is absorbed by a beam dump behind the detector.

## Methods

These methods are expanded from the previous descriptions in ref. 5.

### Sample preparation

Carboxysomes were purified from Halothiobacillus neapolitanus 15147 cells. Cells were broken up by sonication and the debris pelleted at 12,000 g. The supernatant containing carboxysomes was centrifuged at 40,000 g and the pellet was re-suspended in a 1:1 mixture of TEMB buffer (10 mM Tris-HCl, 10 mM MgCl_2_, 20 mM NaHCO_3_, 1 mM EDTA, pH 8.0) and B-PERII (Thermo Scientific). The centrifugation and resuspension steps were repeated twice. The final pellet was resuspended in TEMB and filtered through a 0.2 μm membrane. Carboxysomes were transferred to 20 mM ammonium acetate (pH 7.5), using a PD MiniTrap G-25 column (GE Healthcare), shortly before experiments at the LCLS.

### Injection

An aerosol injector^9^ designed by our group in Uppsala^4,5^ was used to introduce free-flying particles into the pulse train of the X-ray laser at a reduced pressure. Purified carboxysomes were transferred into a volatile buffer (20 mM ammonium acetate, pH 7.5) and were aerosolised with helium, using a gas dynamic virtual nozzle^10^. The sample consumption was 2–4 μlmin^−1^ from a solution of 1.2×10^11^ particles per ml. The aerosol entered the injector via an inlet nozzle coupled to a skimmer. Excess nebulising gas was pumped away at this stage. The concentrated aerosol passed through a relaxation chamber from where the adiabatically cooled particles entered an aerodynamic lens^4,11,12^. The injected sample volume was 36 μl.

### Diffraction

The experiment was carried out with the CAMP instrument^13,14^ at the Atomic, Molecular and Optics (AMO) end-station^15,16^ of the Linac Coherent Light Source^17^ (LCLS). A sketch of the experimental set up with the most important parameters is depicted in Fig. 1c. LCLS delivered X-rays at 1.10 keV (1.13 nm wavelength) in pulses of 120 fs full-duration at half maximum. For the photon energy the band width was 1% and the standard deviation between shots was 0.002 keV. The X-ray beam was focused to a spot of about 5 μm full-width at half maximum, giving about 6.8×10^10^ photons per μm^2^ in the center of the beam, assuming a Gaussian beam profile. The particle beam with a diameter of about 15 μm and a particle flux of 10^8^ particles per minute was intersected with the X-ray laser beam. Diffraction patterns were recorded with a pnCCD area detector^14^ that was positioned at a distance of 741 mm from the interaction region. The detector consists of two panels with 512×1,024 pixels each. The direct beam passes through a gap of 3.7 mm between the panels. The edge length of a single pixel is 75 μm. The detector was operated at low gain with high-charge handling capacity. Seven analog-to-digital units (ADU) measure one photon. The quantum efficiency of the detector at the given photon energy is 0.88. LCLS and the pnCCD detectors were running at 120 Hz and data were recorded over 12 min non-stop.

### Data pre-processing

Data were pre-processed with the Cheetah software package^18^. The 'pedestal-corrected raw data' consist of minimally processed data with dark frame subtraction. Electronic background was removed by subtraction of the average value of 4,834 dark exposures that were recorded prior to the 12 minute-long measurement. The generation of 'pre-processed data' includes labelling and masking of pixels, compensation of detector artefacts, pixel arrangement according to the experimental geometry, persistent photon background subtraction and hit finding. Defective pixels and hot pixels were masked out. Pixels that measured high signal had a non-linear detector response, and were adjusted with an empirical formula derived from measurements with known illumination. Persistent contributions of the photon background were suppressed by subtracting the median pixel values gathered from the ‘misses’ (i.e., non-hits). Pixels that were exposed to a highly fluctuating photon background (standard deviation greater than 3 photons) were masked out. The 'pre-processed data' is immediately useable with minimum additional effort.

### Hit finding

**Figure 2:**
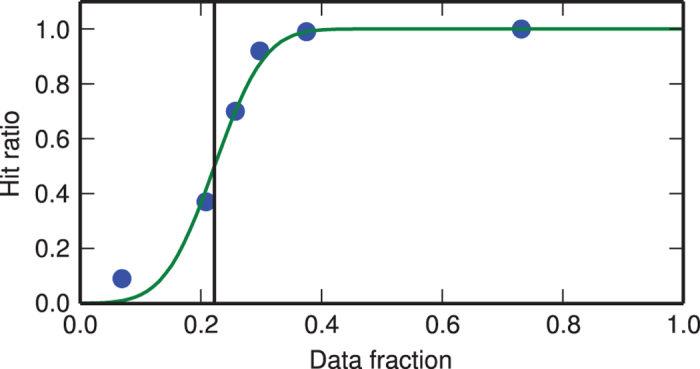
Determination of the threshold for hit finding. Diffraction patterns were sorted according to their number of ‘lit’ pixels in increasing order. Blue data points indicate the manually determined hit ratio for a sequence of 100 consecutive patterns in the sorted array. An error function (green line) approximates the data points. We define the hit threshold (black vertical line) at its inflection point. This corresponds to 400 ‘lit’ pixels and an overall hit ratio of 79%.

A down-sampled pixel was considered ‘lit’ if it contained more than 36 photons. Patterns in which at least 400 8-by-8 down-sampled pixels were lit were considered hits. For defining this threshold diffraction patterns were sorted according to the number of ‘lit’ pixels. Sequences of 100 diffraction patterns in this sorted array were manually inspected and the hit ratios in these subsets were determined (see Fig. 2). The error function was fitted to the data points and the hit threshold was set to the number of lit pixels at the inflection point.

## Data Records

Extracted raw and pre-processed diffraction data are saved to the file *amo55912-r0121.cxi* in the Hierarchical Data Format version 5 (HDF5, http://hdfgroup.org). The data layout is documented at the Coherent X-ray Imaging Data Bank^19^ (CXIDB). The three main data sets are described in Table 1. Data set */entry_1/data_1/data* contains the raw data. Pre-processed data are provided in data set */entry_1/image_1/data*. The arrangement of the 2-dimensional data array corresponds to the true physical arrangement, which results in the slightly larger array size compared to the raw data. Due to the high sampling ratio of the diffraction patterns we also provide the data set */entry_1/image_2/data*, which contains pre-processed frames that were down-sampled 4 by 4.

All files of data record 1 are listed in Table 2. In addition to extracted and pre-processed data we also provide the original files in the eXtended Tagged Container (XTC) format that contain all data recorded during the experiment. We provide also the calibration and configuration files that were used to extract data from the XTC files with the *Cheetah* software package^18^ (git commit 43b4cf5).

The data is deposited on the CXIDB under entry ID 25 (Data Citation 1).

## Technical Validation

**Figure 3:**
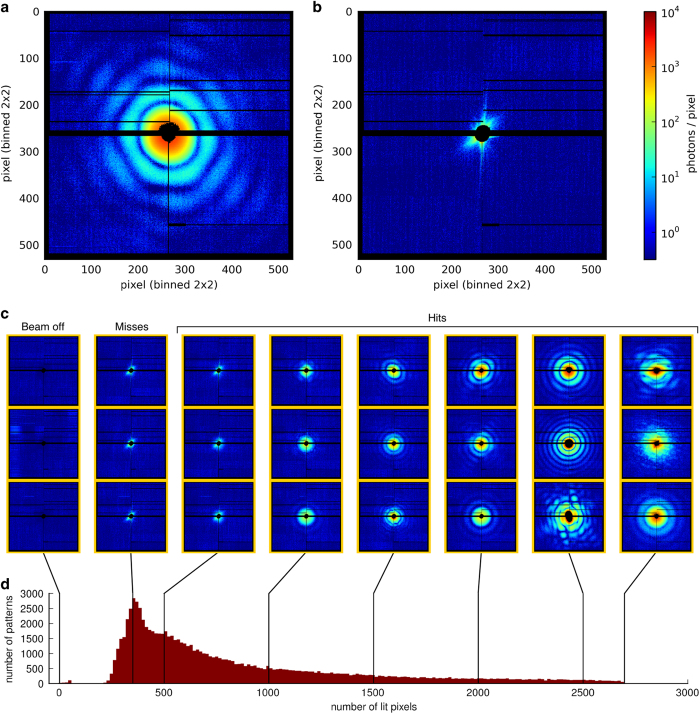
Diffraction images. (**a**) Single-particle diffraction pattern (persistent photon background subtracted). (**b**) Detector read out when no particle was in the beam (no subtraction of the persistent photon background applied). Black regions in the diffraction images indicate missing data. (**c**) Sets of diffraction patterns with similar numbers of lit pixels. (**d**) Histogram of the signal strength from all recorded diffraction patterns.

We show in Fig. 3a the diffraction pattern of a single carboxysome and in Fig. 3b a detector read out from a blank shot that demonstrates high ratio of signal to noise in the data. In Fig. 3c we show a subset of diffraction patterns and the distribution of the brightness of the diffraction patterns. We find that from about 87,000 recorded exposures about 70,000 were hits on at least one particle. Most hits were faint. In the size domain expected for our carboxysomes (100–130 nm) many objects showed straight edges, resembling icosahedra. The predominant fraction of objects in the data set are round. Such round objects are probably some other material of similar density; carboxysomes were purified on the basis of their size and density and impurities can be expected. Nevertheless, we can separate the different structures from each other computationally^5^.

**Figure 4:**
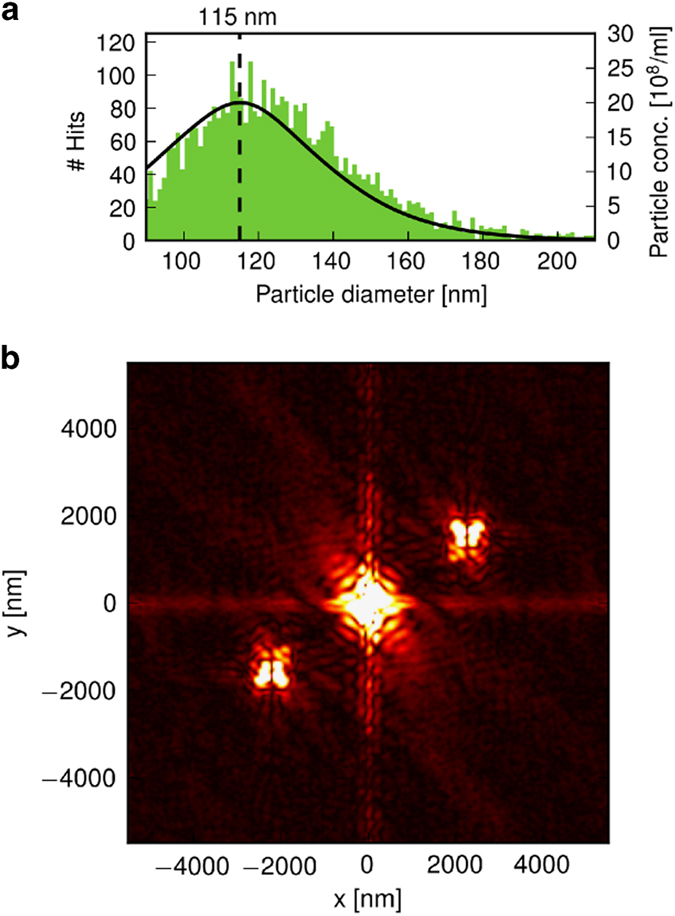
Pattern classification. Figure adapted from ref. 5. (**a**) Size distribution as measured in solution by nano particle tracking analysis (solid black line) and the reconstructed size distribution from the diffraction patterns (green histogram). (**b**) The autocorrelation function with two cross terms indicate the presence of two particles in the field of view.

A first analysis on these data^5^ demonstrated sorting of the diffraction patterns and automatic reconstruction of projection images. The analysis reproduced the size distribution observed in solution (Fig. 4a). In the reconstructions of projection images from individual diffraction patterns phases were retrieved up to a full-period resolution of 18.2 nm. Multiple hits can be discerned from single hits from the autocorrelation function (Fig. 4b).

**Figure 5:**
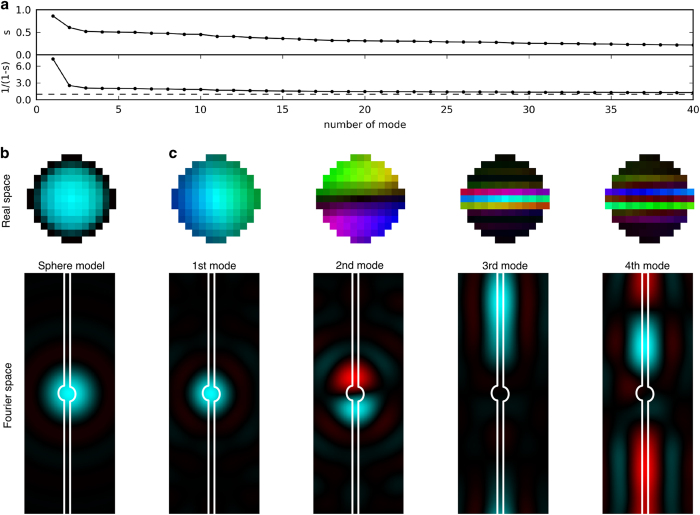
Missing mode analysis. (**a**) Top: Singular values *s* for the first 39 modes that are least constrained by the support constraint and the measured amplitudes. Bottom: For the same modes we plot the theoretical amplification factor 1/(1-*s*) of the noise level that corresponds to the effect from missing data. (**b**) Real space and Fourier space image of a sphere model. (**c**) Real and Fourier space images of the four least constrained modes. The hue corresponds to the phase and the brightness to the amplitude of each pixel. The area between the white lines outlines the missing data region.

Figure 5 shows the effect of the missing data regions. This was estimated for 115-nm-sized particles with a missing mode analysis tool^4,20^. The four least constrained modes have singular values of 0.862, 0.606, 0.520 and 0.509 respectively (Fig. 5a, top). Assuming uniform noise the missing data corresponds to an increase of the noise level by a factor of 7.27, 2.54, 2.08, and 2.04 for the respective modes (Fig. 5a, bottom). Due to the fact that these least constrained modes are most prominent in the centre of the pattern (Fig. 5b) where the signal-to-noise ratio is high suggests that the effect of the missing data is negligible.

## Additional Information

**How to cite this article:** Hantke, M. F. *et al.* A data set from flash X-ray imaging of carboxysomes. *Sci. Data* 3:160061 doi: 10.1038/sdata.2016.61 (2016).

## Supplementary Material



## Figures and Tables

**Table 1:** Data sets of diffraction data.

**Name of data set**	**Description**	**Unit**	**Down-sampling**	**Shape**
/entry_1/data_1/raw/data	Pedestal-corrected raw data	ADU	—	87196×1024×1024
/entry_1/image_1/data	Pre-processed data	Photon/pixel	—	87196×1062×1062
/entry_1/image_2/data	Pre-processed data	Photon/pixel	4 by 4	87196×266×266
This table lists the data sets of extracted and pre-processed diffraction data in the file *amo55912-r0121.cxi*.				

**Table 2:** Files.

**Description**	**Filename**
Extracted and pre-processed diffraction data	amo55912-r0121.cxi
Raw data	e190-r0121-s01-c00.xtc
	e190-r0121-s02-c00.xtc
	e190-r0121-s03-c00.xtc
	e190-r0121-s01-c01.xtc
	e190-r0121-s02-c01.xtc
	e190-r0121-s03-c01.xtc
Calibration data	pnCCD-gaincal-to-photons.h5
	pnCCD-badpixelmask.h5
	pnCCD-metrology.h5
	r0120-pnCCD-darkcal.h5
Configuration files for pre-processing	cheetah.ini
	psana.cfg
This table lists the files associated with data record 1.	
